# Targeting Adiponectin in Breast Cancer

**DOI:** 10.3390/biomedicines10112958

**Published:** 2022-11-17

**Authors:** Rawan Nehme, Mona Diab-Assaf, Caroline Decombat, Laetitia Delort, Florence Caldefie-Chezet

**Affiliations:** 1 Université Clermont-Auvergne, INRAE, UNH Unité de Nutrition Humaine, CRNH-Auvergne, 63000 Clermont-Ferrand, France; 2 Equipe Tumorigénèse Moléculaire et Pharmacologie Anticancéreuse, Faculté des Sciences II, Université Libanaise Fanar, Beyrouth 1500, Lebanon

**Keywords:** adiponectin, breast cancer, obesity, adiponectin receptor agonists, molecular pathways, adiponectin-focused therapeutics

## Abstract

Obesity and breast cancer are two major health issues that could be categorized as sincere threats to human health. In the last few decades, the relationship between obesity and cancer has been well established and extensively investigated. There is strong evidence that overweight and obesity increase the risk of postmenopausal breast cancer, and adipokines are the central players in this relationship. Produced and secreted predominantly by white adipose tissue, adiponectin is a bioactive molecule that exhibits numerous protective effects and is considered the guardian angel of adipokine. In the obesity–cancer relationship, more and more evidence shows that adiponectin may prevent and protect individuals from developing breast cancer. Recently, several updates have been published on the implication of adiponectin in regulating tumor development, progression, and metastases. In this review, we provide an updated overview of the metabolic signaling linking adiponectin and breast cancer in all its stages. On the other hand, we critically summarize all the available promising candidates that may reactivate these pathways mainly by targeting adiponectin receptors. These molecules could be synthetic small molecules or plant-based proteins. Interestingly, the advances in genomics have made it possible to create peptide sequences that could specifically replace human adiponectin, activate its receptor, and mimic its function. Thus, the obvious anti-cancer activity of adiponectin on breast cancer should be better exploited, and adiponectin must be regarded as a serious biomarker that should be targeted in order to confront this threatening disease.

## 1. Introduction

Almost 50 years have passed since breast cancer-related research started booming, and it is still considered a major public health issue with a large impact on cancer deaths worldwide. Breast cancer is a complex, heterogeneous disease that mostly starts as an abnormal proliferation in the lining cells (epithelium) of the ducts (85%) or lobules (15%) in the glandular tissue of the breast [1,2]. Over time and due to various carcinogenic factors, these primary cancers may progress and invade to develop aggressive metastatic carcinomas [3]. Although this stage is considered incurable, it can be controlled by various new treatment protocols that have led to a significant increase in overall survival and the improvement of the quality of life, thus women with metastatic breast cancer are living longer [4]. Four principal molecular subtypes of invasive breast cancer are characterized according to the expression of the estrogen receptor (ER) and progesterone receptor (PR), and the overexpression of the human epidermal growth factor receptor 2 (HER2). Almost 80% of breast cancer patients belong to the Luminal A (ER+ and/or PR+, HER2−) and Luminal B (ER+ and/or PR+, HER2+) subgroups, which highlight the major role of the hormonal receptors in this disease [5]. In 2020, breast cancer ranked as the most commonly diagnosed cancer among 36 cancer types and it surpassed lung cancer in 185 countries [6] with a total of 2.3 million new cases, which represent 11.7% of total cancers worldwide [7]. Despite the huge progress in breast cancer management and the development of several novel targeted therapies, the number of breast-cancer-related deaths remains large, with almost 685,000 deaths in 2020, which correspond to 16% of cancer deaths in women, and the number is predicted to increase to more than 1 million deaths per year by 2040 [8]. Although the mortality rate in developed countries and developing countries is not comparable, the burden of breast cancer mortality is substantial and rising worldwide, which imposes a persistent need to identify new and specific biomarkers that will lead to the development of novel and more effective targeted therapies to increase survival rates. The major risk factors related to breast cancer over the last five years could be summarized by age, family history, menopausal status, lifestyle, genetic factors, and obesity [9,10].

Obesity, defined as a body mass index (BMI) above 30 kg/m^2^, has been recognized as one of the top ten threats to human health by the world health organization (WHO) [11]. It is a complex, multifactorial disorder characterized by an abnormal increase in the adipose tissue, representing one of the most important endocrine glands in the body with a high metabolic activity that could modify the global metabolic state and induce several pathological conditions [12,13,14]. Obesity affects more than 1 billion people worldwide according to the WHO, and the trend seems to be increasing [15,16]. A higher prevalence of obesity was observed in women, and data show that on a global level, the percentage of women suffering from severe obesity is double compared to men. Thus, this gender inequality places emphasis on the link between obesity and physiological sex differences, especially woman-specific hormones. Consequently, ovarian hormones and mainly estrogen have been extensively studied and are shown to influence energy intake (eating), energy expenditure, and the metabolic function of adipose tissue [17]. In this context, several epidemiological studies have shown that abdominal obesity is present in two-thirds of women, especially in postmenopause where the rate of fat gain doubles and lean body mass declines mainly due to acute hormonal changes [18,19].

Mounting clinical evidence now links obesity to a higher risk of at least 15 types of cancer including breast cancer in postmenopausal women where almost 7% of all menopausal breast cancer is attributed to overweight or obesity [15,20]. A very recent multi-center cohort study showed that in breast cancer patients, a higher BMI was associated with a 4% increase in the risk of death for each 1 kg/m^2^ above the mean BMI of the cohort [21]. Obesity may also worsen several aspects of cancer, including higher rates of cancer progression and recurrence, reduced progression-free survival, and increased mortality [16,22]. As most of the breast is covered by adipose tissue, from the collarbone to the underarm and around the center of the ribcage [23], the interplay between adipose tissue and breast cancer cells has been extensively investigated. Excessive estrogen production by adipose tissue is one of the most important molecular mechanisms linking obesity to breast cancer [24]. In postmenopause, the estrogen level in obese women tends to be 50–100% higher than in women with normal weight, which will lead to a greater risk of developing breast cancer [25].

In addition, obesity has been shown to be associated with chronic low-grade inflammation and oxidative stress, along with abnormal variations in the level of biologically active adipokines [26], which could be considered the main mechanisms through which obesity affects breast cancer development. The inflammatory adipose microenvironment of the breast tumor includes more than 600 adipocyte-enriched secretory factors, produced by the adipocytes together with surrounding resident cells such as preadipocytes, fibroblasts, and macrophages, as well as the extracellular matrix, playing a crucial role in cancer development, progression, and metastasis [27].

Most of these secretory factors are adipokines, described as bioactive polypeptides with a range of pro-inflammatory and anti-inflammatory effects. Adipokines, including adiponectin, are becoming more and more important regarding their use in the diagnostic evaluation and treatment of breast cancer [14]. Initially, leptin was the first adipokine associated with obesity and thus it was the most studied factor in regard to the link between obesity and breast cancer risk. The search for the role of leptin in the etiology of breast cancer dates to 1999 [28]. Adiponectin was then shown to have the complete opposite effect of leptin on breast cancer and thus, consecutively gained the attention of several research groups. With its unique biological functions, adiponectin has been considered one of the most important anti-inflammatory and protective agents against obesity-related pathologies [29]. The current review examines the major role of adiponectin as a suppressive agent against breast carcinogenesis and tumor progression, invasion, and metastasis. Special emphasis is placed on the mechanisms and signaling pathways linking adiponectin and breast cancer development and survival. Furthermore, we focus on adiponectin and its signaling axes as potential targets for breast-cancer-specific drugs.

## 2. Adiponectin and Adiponectin Receptors

Adiponectin, one of the most important members of the adipocytokine family [30,31] secreted by adipose tissue, was first isolated in 1995 by Scherer et al. from the mouse adipocyte cell line 3T3-L1 and named Acrp30 (adipocyte complement-related protein of 30-kDa). This discovery was confirmed, almost simultaneously, by three other research groups, Hu et al., Maeda et al., and Nakano et al., in 1996, and Adiponectin was named AdipoQ, apM1 (a product of the most abundant gene of adipose, transcript-1), and GBP28 (gelatin-binding protein-28), respectively [29,32,33]. The final name ‘Adiponectin’ was given by Arita et al. three years later. Adiponectin is a 30-kDa glycoprotein that contains 247 amino acids in mice and 244 amino acids in humans, with 83% homology between them [32]. It belongs to the expanding C1q/TNF family of proteins and is produced as a single subunit that oligomerizes to form at least three distinct and stable isoforms: The trimer, which is the low-molecular-weight (LMW) isoform, the hexamer also called the middle-molecular-weight (MMW) isoform, and the multimer known as the high-molecular-weight (HMW) isoform, which is the most prevalent and biologically active form found in plasma [33,34,35]. Another cleaved globular isoform is also found in different tissues and in circulation. The production of this form was confirmed in 2004 by Waki et al. who demonstrated that the globular isoform is formed through the cleavage of the full-length adiponectin at the amino-terminal collagenous domain by leukocyte elastase, a protease secreted by activated monocytes and neutrophils [30,36,37].

Adiponectin is mainly secreted by white adipose tissue and also, to a limited extent, by the bone marrow, heart, liver, skeletal muscle, and central nervous system [20]. Serum adiponectin levels are inversely correlated with BMI, normally ranging between 2 and 20 µg/mL in people with a BMI over 30 and between 5 and 37 µg/mL in people with a BMI less than 25 [36,38,39]. Thus, adiponectin levels are found to be low in obese people who generally have elevated visceral fat, where its expression is lower than in subcutaneous fat [37].

This adipokine participates in a variety of pathophysiological processes by affecting its target tissues through three ubiquitous surface membrane receptors: The two classical receptors AdipoR1 and AdipoR2 that were first described in 2003 by Yamauchi et al., and one receptor similar to the cadherin family known as T-cadherin or CDH13 that was identified as a potential third adiponectin receptor by Hug et al. in 2004 [32,40]. AdipoR1 is mainly expressed in skeletal muscle, synovial fibroblasts, and endothelial cells, while AdipoR2 is mainly expressed in the liver. The two receptors share a protein sequence that is 67% identical with a 95% shared identity between humans and mice. T-cadherin is mainly expressed in vascular endothelial cells and smooth muscle and has been shown to play a crucial role in cardiovascular protection. The three receptors are expressed in healthy cells and several cancerous tissues including prostate, lung, gastric, and breast cancers. The expression of AdipoR1/R2 is altered by insulin levels so it increases with fasting and physical activities and decreases with eating and obesity [41].

For decades, adiponectin was known as the “Guardian angel” adipocytokine [29,42] since it has been shown to play a central role in preventing and protecting against the development of multiple disorders related to obesity, especially in metabolic syndromes, diabetes, cardiovascular diseases, inflammation, and cancers. Adiponectin has pleiotropic protective and beneficial functions in the central nervous system [43], liver [44], muscles [45], heart [46], bone [47], eye [48], skin [49], and kidney [50,51,52], including promoting insulin sensitivity, controlling whole-body energy by regulating the fat-burning process, glucose intake, and lipid metabolism, and the antioxidant, anti-inflammatory, antiapoptotic, and vasodilatory properties [53,54,55]. Its expression is altered in different pathologies including metabolic syndromes, diabetes mellitus, cardiovascular diseases, Alzheimer’s disease, and several malignancies, mainly uterine and invasive breast cancer. When binding to its receptors, adiponectin modulates a range of intracellular signaling pathways, mainly by activating the adenosine monophosphate-activated protein kinase (AMPK) pathway predominantly through AdipoR1 and peroxisome proliferator-activated receptor alpha (PPAR-α) through AdipoR2. AMPK, in turn, modulates mitogen-activated protein kinase (p38 MAPK), phosphatidylinositol 3-kinase (PI3K), protein kinase B (Akt), wingless-related integration site (Wnt)/β-catenin, mammalian target of rapamycin (mTOR), nuclear factor-κB (NF-κB), c-Jun N-terminal kinase (JNK), and signal transducer and activator of transcription (STAT3), pathways involved not only in cellular metabolism but also in cell proliferation and apoptosis [56].

## 3. The Involvement of Adiponectin in Breast Cancer

**Figure 1:**
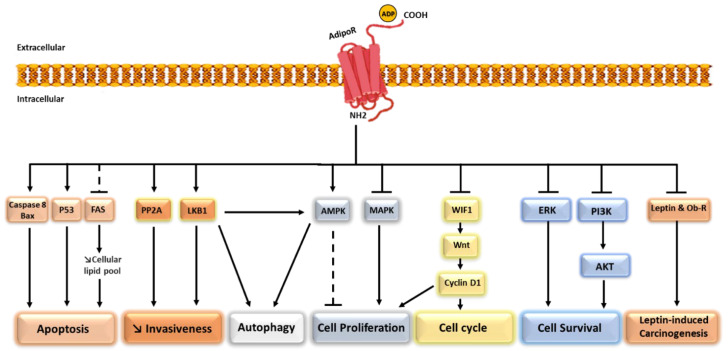
Potent anti-cancer activities of adiponectin by modulating a wide range of signaling pathways. Adiponectin binds to AdipoR1/2 and (1) inhibits cell proliferation by activating AMKP and inhibiting MAKP, (2) induces cell cycle arrest by indirect inhibition of Cyclin D1 through the Wnt pathway, (3) inhibits invasiveness by activating LKB1 and PP2A, (4) induces apoptosis by activating Bax, Caspase 8, and P53 and decreasing the cellular lipid pool through the inhibition of FAS, (5) induces autophagy through LKB1 and AMKP, (6) decreases cell survival through the inhibition of ERK and PI3K, and finally, (7) inhibits leptin-induced carcinogenesis by decreasing the expression of Leptin and the Leptin receptor. ADP: Adiponectin, Bax: Bcl-2–associated X, FAS: Fatty acid synthase, PP2A: Protein phosphatase 2A, LKB1: Liver kinase B1, AMPK: Adenosine monophosphate-activated protein kinase, MAPK: Mitogen-activated protein kinase, WIF1: Wnt inhibitory factor-1, Wnt: Wingless-related integration site, ERK: Extracellular signal-regulated kinase, PI3K: Phosphatidylinositol 3-kinase, Akt: Protein kinase B, Ob-R: Leptin receptor.

For years, overweight and obesity associated with chronic low-grade inflammation have been considered direct, well-recognized risk factors for breast cancer development and recurrence mainly in postmenopause [57]. Thus, the potential role of adipocytes and their secretion in the regulation of breast carcinogenesis has been extensively investigated. In this context, multiple in vitro and in vivo studies have demonstrated the key role of several adipokines including adiponectin in neoplastic progression [20]. Adiponectin exhibits a tumor-suppressive role and has been considered a potent anti-cancer factor by affecting multiple intracellular signaling pathways such as cell proliferation, growth, survival, invasion, and metastasis [37]. The various signaling pathways implicated in adiponectin effects on breast cancer cells are detailed in Figure 1.

### 3.1. Epidemiological Studies

Serum levels of several adipokines, mainly leptin and adiponectin, have been intriguingly associated with an increased risk of breast cancer in multiple studies and meta-analyses. Seven meta-analyses were published between 2013 and 2019 to assess the relationship between serum adiponectin levels and the risk of breast cancer [58,59,60,61,62,63,64]. The overall results confirmed that postmenopausal breast cancer patients had significantly lower serum adiponectin levels and that lower circulating adiponectin was associated with a higher risk of breast cancer development meaning that adiponectin has a protective effect against this cancer. In a meta-analysis published in 2018 by Li Gu et al., the analysis of thirty-one studies investigating the role of adiponectin levels in breast cancer risk suggested a significant decrease in serum adiponectin in breast cancer patients [59]. In another meta-analysis study published in 2017 [58] including 119 studies with 12,301 breast cancer cases and 12,805 controls, Yu Gui et al. found that decreased circulating adiponectin levels and increased concentrations of leptin were significantly associated with increased breast cancer risk. Interestingly, while the individual leptin and adiponectin serum concentrations grabbed the attention of most research groups, other researchers were more attracted by the adiponectin/leptin ratio that was reported to be reduced in women with breast cancer, suggesting that the alteration of this ratio could protect against the disease [65]. Nevertheless, in a clinical trial including 83 patients with stage I-III breast cancer conducted by He et al., the adiponectin level of the participants was measured in 2008 and a follow-up was performed among the surviving patients after 10 years [66], and the results showed that adiponectin levels may be used as a predictor for the survival rates and to determine the patients that require more aggressive treatment.

### 3.2. Role in Tumorigenesis

In contrast to a large number of adipokines, adiponectin has an inhibitory effect on breast tumorigenesis. Adiponectin and its interacting receptors can regulate several intracellular signaling pathways leading to a suppressive effect on carcinogenesis. A large body of in vitro and animal model data support the role of adiponectin as a negative regulator of breast cancer development. In an in vivo study, the supplementation of exogenous adiponectin or its overexpression through adenovirus transduction prior to a xenograft significantly reduces the mammary tumorigenesis of MDA-MB-231 cells in female nude mice [67]. Furthermore, adiponectin inhibits the Wnt and Akt pathways by increasing Wnt inhibitory factor-1 (WIF1) in MDA-MB-231 cells leading to a tumor-suppressive effect [68]. Additionally, further in vivo study using MMTV-polyomavirus middle T antigen transgenic mice with reduced adiponectin expression showed that insufficient adiponectin was associated with early mammary tumorigenesis by activating PI3K/Akt signaling pathway and inhibiting phosphatase and tensin homolog (PTEN) activity [69]. A clinical study that included 25 breast cancer patients with hereditary breast cancer syndrome and 38 healthy relatives showed that in the healthy group, the mutated BRCA carriers have significantly higher adiponectin levels than BRCA wild-type healthy subjects, which confirms that obesity is an important risk factor for hereditary breast cancer and suggests that adiponectin has exhibited a suppressive effect on carcinogenesis in hereditary breast cancer [70]. However, controversial data have been found in ER-positive breast cancer, where the effect of adiponectin seems to be divergent. Several studies have shown that adiponectin could induce cell growth in ER-positive breast cancer through the MAPK activating signaling pathway, thus behaving as a growth factor in ERα-positive breast cancer cells, which suggested the importance of ER status in breast tumorigenesis and the capacity of this pathway to modulate the effect of adiponectin on cell metabolism [71]. On the other hand, other research groups demonstrated an anti-proliferative effect of adiponectin on MCF-7 cells, which are ERα-positive breast cancer cells [72]. Thus, more comprehensive investigations are needed in this regard.

### 3.3. Role in Tumor Progression

Adiponectin inhibits breast cancer progression through several mechanisms that include the inhibition of cellular proliferation in addition to the promotion of apoptosis and cytotoxic autophagy. A recent study by Chung et al. showed that adiponectin induces autophagic cell death in breast cancer in vitro and in vivo, through Serine/threonine kinase 11 (STK11), also known as liver kinase B1 (LKB1)-mediated activation of the AMPK signaling pathway [73]. Other results showed that adiponectin significantly decreased the proliferation of MDA-MB-231 and T47D [67,74], which are two human breast cancer cell lines, and the inhibitory role resulted from the induction of apoptosis coupled with the arrest of the cell cycle at the G0-G1 phase through the indirect suppression of cyclin D1 expression [67]. This direct growth inhibitory effect of adiponectin is mediated mainly by AdipoR1 and not AdipoR2 since treatment with small interference RNA against AdipoR1 significantly reduced the growth inhibition in both cell lines [74]. The antiproliferative effect of adiponectin was also found in other cell lines including MCF-7 where it is involved in the induction of cell apoptosis through the activation of two key enzymes (caspase 8 and caspase 1) and inhibition of the cell cycle [75]. Thus the inhibitory effect of adiponectin was clearly demonstrated on the estrogen-insensitive breast epithelial cancer cell line MDA-MB-231 and on the estrogen-sensitive breast cancer cell line MCF-7 [76], with the difference that ER-positive cells were inhibited at lower adiponectin concentrations than ER-negative cells [77]. In addition to that, the treatment of MCF-7 cells with adiponectin significantly reduces the mRNA of *leptin* and leptin receptors (*Ob-R*) and other genes involved in cell cycle regulation with known growth inhibitory or apoptotic functions, meaning that it inhibits the leptin-induced cell proliferation [72]. In the same concept, an in vivo study showed that adiponectin treatment inhibits leptin-induced mammary tumorigenesis in nude mice by suppressing two canonical signaling molecules, extracellular signal-regulated kinase (ERK) and Akt [42]. Furthermore, a recent study showed that globular adiponectin induces tumor suppression by decreasing the cellular lipid pool, mainly through the inhibition of fatty acid synthase (FAS) and the activation of Sirtuin 1 (SIRT-1), which will promote apoptosis in vivo and in vitro [78].

### 3.4. Role in Metastasis

To achieve metastasis, cancer cells are supposed to alter their microenvironment in order to gain the ability to invade and migrate to reach circulation and then survive in the circulation and exit into new permissive organ sites to colonize distant organs. Understanding this metastatic process is crucial in cancer research because the metastatic potential of tumor cells is linked to a poor survival rate and is considered the major cause of mortality from solid cancers [79]. Thus, molecules in the breast cancer microenvironment, including the adipokines secreted by white adipocytes present in breast tissue, may directly or indirectly affect the evolution of cancer cells toward the metastatic process. Adiponectin, with all its beneficial effects, was supposed to play an important role in regulating breast cancer metastasis. The effect of adiponectin on metastasis was first described in 2009, where several research groups showed that adiponectin inhibits the adhesion, migration, and invasion of breast cancer cells mainly by increasing the expression of *LKB1*, a tumor-suppressor gene that activates the AMPK–S6K axis [80,81]. Furthermore, Kim et al. showed in the same year that adiponectin reduces the invasiveness of MDA-MB-231 breast cancer cells by activating the tumor suppressor protein phosphatase 2A (PP2A) [69,82]. Adiponectin was also shown to effectively inhibit leptin-stimulated migration and invasion of breast cancer cells [42]. In a case-control study, Kang et al. showed that in patients with adiponectin levels below the median, the number of lymph node metastases was significantly increased [83]. However, controversial data have been found on the effect of a specific form of adiponectin, which is the globular isoform, on breast cancer metastasis. A review published in 2014 highlighted a possible positive role of globular adiponectin in the breast cancer metastatic process [30]. The author’s conclusion was based on a bibliographic review of the role of globular adiponectin in several other cancers without direct robust evidence or scientific research in breast cancer. The globular adiponectin, which is a cleaved form of adiponectin, was shown to play a negative role by increasing cellular proliferation, migration, angiogenesis, the activation of matrix metalloproteinases, and the production of reactive oxygen species (ROS) [84,85,86]. Two years later, Libby and colleagues investigated the role of globular adiponectin on metastasis for the first time in breast cancer and showed that globular adiponectin might possibly enhance breast cancer invasion, and this impact was partially dependent on autophagic induction [87]. On the other hand, a recent study showed that globular adiponectin inhibits the growth of breast cancer cells by suppressing inflammasome activation partially through AMPK activation [88]. Larger investigational studies concerning this specific form of adiponectin are required to better understand its potentially mediating role.

### 3.5. Role in Treatment Resistance/Response

Several adipokines participate in the complex process leading to treatment resistance in breast cancer. For example, leptin was shown to induce cisplatin, docetaxel, letrozole, and other chemoresistance [37,89] in addition to its ability to induce hormonal therapy resistance mainly through promoting the proliferation of cancer stem cells [90]. Regarding adiponectin, there are very few scientific data evaluating the relationship between the level of adiponectin and the anti-tumor drug efficacy or treatment resistance, except in a clinical study published in 2012 by Macis and colleagues where it was shown that there was no interaction between treatment and adiponectin levels in 235 premenopausal women with breast cancer taking low-dose tamoxifen and fenretinide [91]. More investigations are needed to fully elucidate the impact of adiponectin on breast cancer treatment resistance. In the same context, Ozman and colleagues showed that radiotherapy significantly increases serum adiponectin, and this could be explained by the potential role of adiponectin in the regulation of tissue damage and repair following radiation therapy [92].

## 4. Reactivation of Adiponectin Pathways in Breast Cancer Models

Due to the beneficial effect of adiponectin on several diseases related to obesity, including diabetes, cardiac fibrosis, and a range of cancers, the pharmacological elevation of circulating adiponectin became the main interest of multiple research groups. Hence, after detailing the molecular mechanisms linking adiponectin and breast cancer tumorigenesis, it seems to be very important to identify new, related, promising therapeutic strategies for the management of breast cancer and the reactivation of adiponectin pathways in breast cancer models. The most obvious way to increase adiponectin levels is body weight reduction through calory restriction and exercise. Adiponectin-based therapies are currently unavailable for several reasons, including the heterogeneity of the expressed protein structures and the extreme insolubility of the C-terminal domain [93]. Fortunately, various adiponectin-replacement therapy options, including peptide and small-molecule-based adiponectin receptor activators, have been suggested in order to overcome the limitations of converting the biologically active adiponectin protein into a viable drug. Ten suggested therapies activating adiponectin signaling were detected in the literature, and their details may be found in Table 1.

### 4.1. Adiponectin Receptor Agonists

Since adiponectin has different isoforms and there is debate regarding the beneficial effect of all of them, targeting the adiponectin receptors appears to be one of the best ways to achieve the expected beneficial effect. Several adiponectin receptor agonists were identified and tested in vitro and in vivo.

Osmotin, a plant-derived protein that can be found in most fruits and vegetables, was the first adiponectin receptor agonist discovered and was shown to share structural and functional homology with adiponectin without sharing sequence similarity [110]. In 2005, Narasimhan and colleagues showed that osmotin exhibits antifungal and apoptotic activities through a seven-transmembrane-domain receptor-like polypeptide called PHO36. The research group showed that AdipoR1 and AdipoR2 are the mammalian homologs of PHO36, thus they suggested that osmotin is the homolog of mammalian adiponectin. Additionally, they demonstrated that osmotin activates AMPK, similarly to adiponectin, in C2C12 myocytes via adiponectin receptors [95], thus it mimics human adiponectin function. Recently, a study conducted by Cohen et al. suggested that osmotin could restore endothelial homeostasis in patients with cardiovascular disease and may prevent microvascular endothelial dysfunction [96]. Furthermore, Geetha et al. suggested the anti-cancer activity of osmotin on MDA-MB-231 breast cancer cells, mainly through the production of senescence-associated ROS [94].

ADP355, a ten-residue peptide mimetic (peptidomimetic), is the first designed peptide-based adiponectin receptor agonist derived from the globular domain of adiponectin that can bind to AdipoR1 and AdipoR2 with a better affinity to AdipoR1. ADP355, designed in 2011 by Otvos and colleagues, exhibits the same effect of adiponectin in several conditions including renal and liver fibrosis, prostate and breast cancers, where it increases AMPK and STAT3 phosphorylation in MCF-7 ERα-positive breast cancer cells and inhibits the phosphorylation of ERK1/2 in MDA-MB-231 ERα-negative breast cancer cells [97]. The beneficial effect of this peptidomimetic was also observed in vivo where the intraperitoneal injection of ADP355 decreases tumor size by 31% compared to untreated SCID mice xenotransplanted with MCF-7 cells and shows no toxic properties to normal mice up to 10 mg/kg [97].

Three years later, the same research group developed and tested the second-generation peptide, ADP399, which showed 20-fold improved cellular activity inhibiting MCF-7 cell growth [106]. A second attempt to optimize ADP355 did not show any notably improved cellular activities [111].

In 2017, Ma et al. identified, in silico, a novel AdipoR1 peptide agonist similar to the minimal active site sequence, named Pep70 and showed that it significantly inhibited the proliferation of hepatic stellate cells thereby reducing the fibrotic response [108].

Furthermore, ADP27, a 10-amino acid peptide consisting of the minimal active site of adiponectin, developed by Marangoni et al., showed beneficial effects in the skin fibrosis model but has not been tested on breast cancer models [98].

In 2018, Sayeed et al. identified and characterized ADP-1, which is a 13-residue peptide derived from the collagen domain of adiponectin. ADP-1 showed adiponectin-like activity in vivo and in vitro, which includes the activation of AMPK and the improvement of glucose and fatty acid metabolisms, with significant stability in human serum [109].

In the same year, a novel potent AdipoR1 peptide agonist, BHD-1028, was identified by Kim et al. based on the crystal structure of AdipoR1. This peptide is able to exhibit its biological activity by inducing AMPK phosphorylation at a nanomolar concentration in a mouse myotube model [36].

In parallel with the discovery of these peptidic agonists, other research groups have searched for non-peptidic AdipoR agonist small molecules from natural products or chemical libraries. Arctin, arctigenin, and gramine were identified from a 10,000-member natural product library but failed to identify their activity (even the best hit retains only 3.5 µM activity) [93].

AdipoRon was identified in 2013 from a chemical library by Okada-lwabu and colleagues [112] at the University of Tokyo and has been the subject of much interest and many studies in adiponectin replacement therapies. AdipoRon is an orally active synthetic small molecule that binds and activates both AdipoR1 and AdipoR2 in vitro [112]. AdipoRon exhibits an adiponectin-like effect in multiple health conditions including obesity [112], diabetes [113], cardiac fibrosis [114], neuroinflammation and depression [115], liver inflammation [116], and dermal fibrosis [117], in addition to its anti-proliferative effect against several cancers including pancreatic cancer [100,102], ovarian cancer [103], osteosarcoma [105], and myeloma [104]. AdipoRon, as a natural adiponectin, exhibits its biological effects mainly through the activation of the AMPK signaling pathway.

Another molecule, the 6-C-β-D-glucopyranosyl-(2S,3S)-(+)-5,7,3’,4’-tetrahydroxydihydroflavonol known as GTDF, was discovered and characterized as an orally active adiponectin mimetic by Singh et al. in 2014 [107]. GTDF is a natural analog of the dietary flavonoid quercetin and is an orally bioavailable osteoanabolic compound. It has been shown to induce adiponectin-related signaling and improve metabolic health [107,118].

Tiliroside, a glycosidic flavonoid biosynthesized by several edible plants including raspberry and strawberry, has also been identified with a wide range of beneficial bioactivities [119]. In 2012, a study showed that tiliroside activates adiponectin signaling pathways, mainly AMPK and PPAR-α, thereby ameliorating obesity-induced metabolic disorders [101]. Although tiliroside has been shown to up-regulate the mRNA expression levels of AdipoR1 and AdipoR2, no studies have reported its binding to receptors to date and therefore it cannot be considered a true AdipoR agonist.

### 4.2. Adiponectin-Focused Therapeutic Strategies

Several molecules might serve as adiponectin replacement agents by exerting their biological effect either by activating the adiponectin signaling pathway or by upregulating the expression of adiponectin itself [120]. PPARγ agonists, which mainly include efatutazone [62] and thiazolidinediones (TZDs) and also called glitazones, which are a class of synthetic small molecules and anti-diabetic drugs that enclose rosiglitazone, troglitazone, and pioglitazone [121,122,123,124,125,126], are the main and most studied molecules that could have a potential therapeutic effect by inducing the expression and secretion of adiponectin. Efatutazone, which is a high-affinity PPARγ agonist, was shown to delay the progression of ductal carcinoma in situ to invasive ductal carcinoma in vivo [121]. The administration of these oral hypoglycemic TZDs significantly activates PPARγ, which in turn improves the secretory profile of the adipose tissue and, not surprisingly, significantly increases plasma adiponectin concentrations in humans in a dose- and time-dependent manner, without affecting their body weight [122]. Additional studies showed that rosiglitazone suppresses the migration, invasion, and cell growth of breast cancer cells by inhibiting the leptin signaling pathway, which is mediated by MAPK/STAT3/Akt phosphorylation, suggesting that this PPARγ ligand prevents the stimulatory effect of leptin on estrogen signaling mainly by enhancing the expression of adiponectin [125,127]. Although they are classified as the most effective hypoglycemic treatments, these drugs are accompanied by a series of side effects, mainly cardiovascular toxicity, that have led to their restriction or even withdrawal from the market in several countries [128].

In addition to PPARγ agonists, there is emerging evidence that the potent long-acting glucagon-like peptide-1 (GLP-1) receptor agonist liraglutide exhibits action against adiponectin that mimics its effects in vitro and in vivo [129,130]. Liraglutide is an anti-diabetic drug that has been shown to significantly increase plasma adiponectin levels in a dose-dependent manner [131]. Liraglutide has been reported to exhibit anti-proliferative activity on human prostate, pancreatic, and hepatocellular carcinomas. Most importantly, liraglutide significantly inhibits MCF-7 proliferation in an obesity-like microenvironment while increasing the mRNA levels of adiponectin and its receptors [132].

In the same context, the famous anti-diabetic drug, metformin, was described as an adiponectin agonist and has been shown to increase serum adiponectin levels and subsequently copy its effect by regulating adiponectin-related signaling pathways [133,134]. Interestingly, metformin has gained huge attention in cancer treatment, especially in breast cancer, and its potential anti-tumorigenic effect was shown to be mediated through the regulation of several pathways that include the potentiation of adiponectin [135]. In a recent study, Leng et al. demonstrated that metformin significantly increases serum adiponectin levels in overweight/obese pre-menopausal women [136]. A huge number of preclinical studies and more than 50 ongoing and completed clinical trials suggested the repurposing of this well-tolerated, cost-effective drug to be used in early and advanced breast cancer patients, and approval is eagerly awaited [137].

## 5. Conclusions

As breast cancer is the most common cancer and the leading cause of mortality in women worldwide, there is an urgent need to develop new therapies targeting this aggressive malignancy. During the last few years, a growing novel list of personalized therapies has been approved for the treatment of breast cancer including alpelisib targeting PI3K, talazoparib and olaparib that target germline BRCA mutated breast cancer, and abemaciclib as a cell cycle inhibitor for patients with high Ki67 [138]. However, despite all these advancements that have led to a very new era of precision medicine in the management of breast cancer, more than 1800 women die every day from this cancer. Furthermore, when reviewing the ongoing clinical trials, we can find that, apart from Elacestrant, which is a novel oral estrogen degrader, there are no new breast cancer therapies entering human clinical trials according to clinicaltrials.gov (accessed end October 2022). On the other hand, in the preclinical exploratory phase, the impact of obesity on breast cancer has been proven and well-known for a long time, and researchers are trying to investigate this relationship to discover novel targets in the regulation of carcinogenesis. Adiponectin has been demonstrated to be one of the most important molecules linking obesity and breast cancer and has been described as a rising predictive and prognostic biomarker. Despite the huge interest of several research groups in targeting this cytokine since 2005, none of the ten promising adiponectin agonist candidates listed in this review have successfully reached phase I clinical trials as a systemic cancer therapy. Thus, there is a serious need to better evaluate these molecules in order to find the most effective adiponectin replacement candidate that could reach human clinical trials in the near future.

To reach this stage, a huge amount of work remains to be completed, from the identification of the best potential candidate to the optimization of the dose and administration modes and the safety profile. In this regard, three out of the ten molecules detailed in this review have been tested on breast cancer and were found to exhibit significant anti-cancer properties, which could be an encouraging start to further exploit their effects. In recent decades, peptide-based drug candidates have attracted the attention of researchers because of their high specificity and low toxicity, and they have been widely used in cancer treatment over the last few years. The efforts in peptide drug discovery have led to the approval of more than eighty peptide drugs that have entered the market since 1921 [139], and more than 170 peptides are in active clinical development [140]. Compared with small molecules, peptide drugs have higher rates of clinical trial success and lower production costs [141], which will provide an advantage to the peptide adiponectin receptor agonists mentioned in our review, especially AP355, to achieve their final targets in the drug discovery and development process by obtaining approval and entering the therapeutic market. However, it is also important to focus on the fact that these molecules do not specifically target cancer cells to induce the desired human response. Thus, they could have other effects on other off-target cell types, which could include dendritic cells as shown by Tan and colleagues [142], and this would need to be further evaluated in order to achieve the desired outcome without unwanted adverse side effects. On the other hand, and since drug repurposing has recently attracted increasing interest regarding cancer, the repurposing of several anti-diabetic drugs, including metformin, to be used as adiponectin’s replacement treatment seems to be very promising because it will avoid the strict Food and Drug Administration (FDA) regulations [143].

An alternative approach to interventions could be to target obesity and adipose tissue instead of searching for a direct cytotoxic effect on breast cancer cells themselves, which could provide further benefits for women with overweight or obesity suffering from breast cancer. This strategy may lead to several benefits, including minimizing the risk of developing breast cancer, attenuating the aggressivity and the progression of the disease, and reducing the recurrence in overweight and obese breast cancer survivors. Several plant-based bioactive components could serve as potential and safe candidates targeting obesity. The use of these molecules in combination with cancer treatment as a personalized concept could be helpful to eradicate tumors entirely, prevent recurrence, and maintain a good quality of life for these patients.

## Figures and Tables

**Table 1:** List of the main adiponectin receptor agonists found in the literature and their published activities.

Molecule	Structure	Cell Type/Model	Year	Study Type	Pathways	Function	Approxi. IC50	Ref.
Osmotin	Plant-derived protein	Myocytes Human arterioles Breast cancer	2005	In vitro	AMPK	AMPK activation Restore endothelial homeostasis Induce ROS-associated senescence	0.3 μM 200 µg/mL	[94,95,96]
ADP355	10-residue peptide	Prostate, breast cancers, fibroblast	2011	In vitro In vivo	AMPK and STAT3 ERK1/2 AKT	Renal and Liver anti-fibrotic effect Inhibit prostate and breast cancer cell proliferation	25 nM 5 mg/kg	[97,98,99,100]
Tiliroside	Glycosidic flavonoid	obese-diabetic KK-A(y) mice	2012	In vivo	AMPK PPARα	Ameliorating obesity-induced metabolic disorders	100 mg/kg	[101]
AdipoRon	Synthetic small-molecule	Pancreatic and ovarian carcinomas, osteosarcoma, myeloma	2013	In vitro	AMPK	Inhibiting pancreatic, ovarian, osteosarcoma and myeloma cell growth AMPK activation	5 μm	[102,103,104,105]
ADP399	Dimer of ADP355	Breast	2014	In vitro	AMPK	Inhibiting breast cancer cell growth	10 nM	[106]
GTDF	Analog of quercetin	Myoblasts, pre-adipocytes and epithelial cells	2014	In vitro In vivo	AMPK, ACC, P38	Enhanced glucose uptake Improve metabolic health	10 nM 10 mg/kg	[107]
Pep70	7-residue Peptide	Hepatic stellate cells	2017	In vitro		Anti-fibrotic	10 μM	[108]
ADP27	10-residue peptide	Skin fibrosis model	2017	In vitro In vivo	AMPK	Anti-fibrotic		[98]
ADP-1	13-residue peptide	Skeletal muscle cells	2018	In vitro In vivo	AMPK	Enhanced glucose uptake AMPK activation	14.3 μg/mL 30 mg/kg	[109]
BHD-1028	15-residue Peptide	Myotube model	2018	In vivo	AMPK	AMPK activation	<800 nM	[36]

## Data Availability

Not applicable.

## References

[1] Sun Y.-S., Zhao Z., Yang Z.-N., Xu F., Lu H.-J., Zhu Z.-Y., Shi W., Jiang J., Yao P.-P., Zhu H.-P. (2017). Risk Factors and Preventions of Breast Cancer. Int. J. Biol. Sci..

[2] Nagini S. (2017). Breast Cancer: Current Molecular Therapeutic Targets and New Players. Anticancer Agents Med. Chem..

[3] Kwapisz D. (2019). Oligometastatic Breast Cancer. Breast Cancer Tokyo Jpn..

[4] Tosello G., Torloni M.R., Mota B.S., Neeman T., Riera R. (2018). Breast Surgery for Metastatic Breast Cancer. Cochrane Database Syst. Rev..

[5] Burguin A., Diorio C., Durocher F. (2021). Breast Cancer Treatments: Updates and New Challenges. J. Pers. Med..

[6] Sung H., Ferlay J., Siegel R.L., Laversanne M., Soerjomataram I., Jemal A., Bray F. (2021). Global Cancer Statistics 2020: GLOBOCAN Estimates of Incidence and Mortality Worldwide for 36 Cancers in 185 Countries. CA. Cancer J. Clin..

[7] Arnold M., Morgan E., Rumgay H., Mafra A., Singh D., Laversanne M., Vignat J., Gralow J.R., Cardoso F., Siesling S. (2022). Current and Future Burden of Breast Cancer: Global Statistics for 2020 and 2040. Breast Off. J. Eur. Soc. Mastology.

[8] Anderson B.O., Ilbawi A.M., Fidarova E., Weiderpass E., Stevens L., Abdel-Wahab M., Mikkelsen B. (2021). The Global Breast Cancer Initiative: A Strategic Collaboration to Strengthen Health Care for Non-Communicable Diseases. Lancet Oncol..

[9] Fakhri N., Chad M.A., Lahkim M., Houari A., Dehbi H., Belmouden A., El Kadmiri N. (2022). Risk Factors for Breast Cancer in Women: An Update Review. Med. Oncol. Northwood Lond. Engl..

[10] Pischon T., Nimptsch K., Pischon T., Nimptsch K. (2016). Obesity and Risk of Cancer: An Introductory Overview. Obesity and Cancer.

[11] Zorena K., Jachimowicz-Duda O., Ślęzak D., Robakowska M., Mrugacz M. (2020). Adipokines and Obesity. Potential Link to Metabolic Disorders and Chronic Complications. Int. J. Mol. Sci..

[12] Parrettini S., Cavallo M., Gaggia F., Calafiore R., Luca G. (2020). Adipokines: A Rainbow of Proteins with Metabolic and Endocrine Functions. Protein Pept. Lett..

[13] Frigolet M.E., Gutiérrez-Aguilar R. (2020). The Colors of Adipose Tissue. Gac. Med. Mex..

[14] Kim D.-S., Scherer P.E. (2021). Obesity, Diabetes, and Increased Cancer Progression. Diabetes Metab. J..

[15] Devericks E.N., Carson M.S., McCullough L.E., Coleman M.F., Hursting S.D. (2022). The Obesity-Breast Cancer Link: A Multidisciplinary Perspective. Cancer Metastasis Rev..

[16] Das M., Webster N.J.G. (2022). Obesity, Cancer Risk, and Time-Restricted Eating. Cancer Metastasis Rev..

[17] Leeners B., Geary N., Tobler P.N., Asarian L. (2017). Ovarian Hormones and Obesity. Hum. Reprod. Update.

[18] da Costa Pimenta W., Santos Brant Rocha J., Prates Caldeira A., Araújo Veloso Popoff D., Maia Santos V., Murça de Souza J.E., Marques M.S., Piana Santos Lima de Oliveira F., Rodrigues Caldeira D.M., Souza Guerra Júnior G.E. (2020). Abdominal Obesity and Association with Sociodemographic, Behavioral and Clinical Data in Climacteric Women Assisted in Primary Care. PLoS ONE.

[19] El Khoudary S.R., Greendale G., Crawford S.L., Avis N.E., Brooks M.M., Thurston R.C., Karvonen-Gutierrez C., Waetjen L.E., Matthews K. (2019). The Menopause Transition and Women’s Health at Midlife: A Progress Report from the Study of Women’s Health Across the Nation (SWAN). Menopause.

[20] Christodoulatos G.S., Spyrou N., Kadillari J., Psallida S., Dalamaga M. (2019). The Role of Adipokines in Breast Cancer: Current Evidence and Perspectives. Curr. Obes. Rep..

[21] Kohls M., Freisling H., Charvat H., Soerjomataram I., Viallon V., Davila-Batista V., Kaaks R., Turzanski-Fortner R., Aleksandrova K., Schulze M.B. (2022). Impact of Cumulative Body Mass Index and Cardiometabolic Diseases on Survival among Patients with Colorectal and Breast Cancer: A Multi-Centre Cohort Study. BMC Cancer.

[22] Petrelli F., Cortellini A., Indini A., Tomasello G., Ghidini M., Nigro O., Salati M., Dottorini L., Iaculli A., Varricchio A. (2021). Association of Obesity With Survival Outcomes in Patients With Cancer: A Systematic Review and Meta-Analysis. JAMA Netw. Open.

[23] Kothari C., Diorio C., Durocher F. (2020). The Importance of Breast Adipose Tissue in Breast Cancer. Int. J. Mol. Sci..

[24] Wang X., Simpson E.R., Brown K.A. (2015). Aromatase Overexpression in Dysfunctional Adipose Tissue Links Obesity to Postmenopausal Breast Cancer. J. Steroid Biochem. Mol. Biol..

[25] Özgöz A., Mutlu Içduygu F., Yükseltürk A., Samli H., Hekimler Öztürk K., Baskan Z., Tütüncü I. (2021). Postmenopausal Estrogen Receptor Positive Breast Cancer and Obesity Associated Gene Variants. EXCLI J..

[26] Kawai T., Autieri M.V., Scalia R. (2021). Adipose Tissue Inflammation and Metabolic Dysfunction in Obesity. Am. J. Physiol. Cell Physiol..

[27] Habanjar O., Diab-Assaf M., Caldefie-Chezet F., Delort L. (2022). The Impact of Obesity, Adipose Tissue, and Tumor Microenvironment on Macrophage Polarization and Metastasis. Biology.

[28] Mantzoros C.S., Bolhke K., Moschos S., Cramer D.W. (1999). Leptin in Relation to Carcinoma in Situ of the Breast: A Study of Pre-Menopausal Cases and Controls. Int. J. Cancer.

[29] Parida S., Siddharth S., Sharma D. (2019). Adiponectin, Obesity, and Cancer: Clash of the Bigwigs in Health and Disease. Int. J. Mol. Sci..

[30] Libby E.F., Frost A.R., Demark-Wahnefried W., Hurst D.R. (2014). Linking Adiponectin and Autophagy in the Regulation of Breast Cancer Metastasis. J. Mol. Med. Berl. Ger..

[31] Achari A.E., Jain S.K. (2017). Adiponectin, a Therapeutic Target for Obesity, Diabetes, and Endothelial Dysfunction. Int. J. Mol. Sci..

[32] Nguyen T.M.D. (2020). Adiponectin: Role in Physiology and Pathophysiology. Int. J. Prev. Med..

[33] Kalkman H.O. (2021). An Explanation for the Adiponectin Paradox. Pharm. Basel Switz..

[34] Wang Z.V., Scherer P.E. (2016). Adiponectin, the Past Two Decades. J. Mol. Cell Biol..

[35] van Andel M., Heijboer A.C., Drent M.L. (2018). Adiponectin and Its Isoforms in Pathophysiology. Adv. Clin. Chem..

[36] Kim S., Lee Y., Kim J.W., Son Y.-J., Ma M.J., Um J.-H., Kim N.D., Min S.H., Kim D.I., Kim B.B. (2018). Discovery of a Novel Potent Peptide Agonist to Adiponectin Receptor 1. PLoS ONE.

[37] Cha Y.J., Koo J.S. (2018). Adipokines as Therapeutic Targets in Breast Cancer Treatment. Expert Opin. Ther. Targets.

[38] Raut P.K., Park P.-H. (2020). Globular Adiponectin Antagonizes Leptin-Induced Growth of Cancer Cells by Modulating Inflammasomes Activation: Critical Role of HO-1 Signaling. Biochem. Pharmacol..

[39] Waki H., Yamauchi T., Kamon J., Kita S., Ito Y., Hada Y., Uchida S., Tsuchida A., Takekawa S., Kadowaki T. (2005). Generation of Globular Fragment of Adiponectin by Leukocyte Elastase Secreted by Monocytic Cell Line THP-1. Endocrinology.

[40] Iwabu M., Okada-Iwabu M., Yamauchi T., Kadowaki T. (2019). Adiponectin/AdipoR Research and Its Implications for Lifestyle-Related Diseases. Front. Cardiovasc. Med..

[41] Tumminia A., Vinciguerra F., Parisi M., Graziano M., Sciacca L., Baratta R., Frittitta L. (2019). Adipose Tissue, Obesity and Adiponectin: Role in Endocrine Cancer Risk. Int. J. Mol. Sci..

[42] Taliaferro-Smith L., Nagalingam A., Knight B.B., Oberlick E., Saxena N.K., Sharma D. (2013). Integral Role of PTP1B in Adiponectin-Mediated Inhibition of Oncogenic Actions of Leptin in Breast Carcinogenesis. Neoplasia.

[43] Bloemer J., Pinky P.D., Govindarajulu M., Hong H., Judd R., Amin R.H., Moore T., Dhanasekaran M., Reed M.N., Suppiramaniam V. (2018). Role of Adiponectin in Central Nervous System Disorders. Neural Plast..

[44] Gamberi T., Magherini F., Modesti A., Fiaschi T. (2018). Adiponectin Signaling Pathways in Liver Diseases. Biomedicines.

[45] Gamberi T., Magherini F., Fiaschi T. (2019). Adiponectin in Myopathies. Int. J. Mol. Sci..

[46] Sharma A., Mah M., Ritchie R.H., De Blasio M.J. (2022). The Adiponectin Signalling Pathway—A Therapeutic Target for the Cardiac Complications of Type 2 Diabetes?. Pharmacol. Ther..

[47] Naot D., Musson D.S., Cornish J. (2017). The Activity of Adiponectin in Bone. Calcif. Tissue Int..

[48] Li H.-Y., Hong X., Cao Q.-Q., So K.-F., Yau S.-Y., So K.-F. (2019). Chapter Eleven—Adiponectin, Exercise and Eye Diseases. International Review of Neurobiology.

[49] Oh J., Lee Y., Oh S.-W., Li T., Shin J., Park S.-H., Lee J. (2022). The Role of Adiponectin in the Skin. Biomol. Ther..

[50] Karamian M., Moossavi M., Hemmati M. (2021). From Diabetes to Renal Aging: The Therapeutic Potential of Adiponectin. J. Physiol. Biochem..

[51] Przybyciński J., Dziedziejko V., Puchałowicz K., Domański L., Pawlik A. (2020). Adiponectin in Chronic Kidney Disease. Int. J. Mol. Sci..

[52] Kökény G., Calvier L., Hansmann G. (2021). PPARγ and TGFβ—Major Regulators of Metabolism, Inflammation, and Fibrosis in the Lungs and Kidneys. Int. J. Mol. Sci..

[53] Ruan H., Dong L.Q. (2016). Adiponectin Signaling and Function in Insulin Target Tissues. J. Mol. Cell Biol..

[54] Esmaili S., Hemmati M., Karamian M. (2020). Physiological Role of Adiponectin in Different Tissues: A Review. Arch. Physiol. Biochem..

[55] Fasshauer M., Blüher M. (2015). Adipokines in Health and Disease. Trends Pharmacol. Sci..

[56] Fang H., Judd R.L. (2018). Adiponectin Regulation and Function. Compr. Physiol..

[57] Avgerinos K.I., Spyrou N., Mantzoros C.S., Dalamaga M. (2019). Obesity and Cancer Risk: Emerging Biological Mechanisms and Perspectives. Metabolism.

[58] Gui Y., Pan Q., Chen X., Xu S., Luo X., Chen L. (2017). The Association between Obesity Related Adipokines and Risk of Breast Cancer: A Meta-Analysis. Oncotarget.

[59] Gu L., Cao C., Fu J., Li Q., Li D.-H., Chen M.-Y. (2018). Serum Adiponectin in Breast Cancer: A Meta-Analysis. Medicine.

[60] Liu L.-Y., Wang M., Ma Z.-B., Yu L.-X., Zhang Q., Gao D.-Z., Wang F., Yu Z.-G. (2013). The Role of Adiponectin in Breast Cancer: A Meta-Analysis. PLoS ONE.

[61] Macis D., Guerrieri-Gonzaga A., Gandini S. (2014). Circulating Adiponectin and Breast Cancer Risk: A Systematic Review and Meta-Analysis. Int. J. Epidemiol..

[62] Yoon Y.S., Kwon A.R., Lee Y.K., Oh S.W. (2019). Circulating Adipokines and Risk of Obesity Related Cancers: A Systematic Review and Meta-Analysis. Obes. Res. Clin. Pract..

[63] Yu Z., Tang S., Ma H., Duan H., Zeng Y. (2019). Association of Serum Adiponectin with Breast Cancer: A Meta-Analysis of 27 Case-Control Studies. Medicine.

[64] Ye J., Jia J., Dong S., Zhang C., Yu S., Li L., Mao C., Wang D., Chen J., Yuan G. (2014). Circulating Adiponectin Levels and the Risk of Breast Cancer: A Meta-Analysis. Eur. J. Cancer Prev..

[65] Cleary M.P., Ray A., Rogozina O.P., Dogan S., Grossmann M.E. (2009). Targeting the Adiponectin:Leptin Ratio for Postmenopausal Breast Cancer Prevention. Front. Biosci..

[66] Güven H.E., Doğan L., Gülçelik M.A., Gülçelik N.E. (2018). Adiponectin: A Predictor for Breast Cancer Survival?. Eur. J. Breast Health.

[67] Wang Y., Lam J.B., Lam K.S.L., Liu J., Lam M.C., Hoo R.L.C., Wu D., Cooper G.J.S., Xu A. (2006). Adiponectin Modulates the Glycogen Synthase Kinase-3beta/Beta-Catenin Signaling Pathway and Attenuates Mammary Tumorigenesis of MDA-MB-231 Cells in Nude Mice. Cancer Res..

[68] Liu J., Lam J.B.B., Chow K.H.M., Xu A., Lam K.S.L., Moon R.T., Wang Y. (2008). Adiponectin Stimulates Wnt Inhibitory Factor-1 Expression through Epigenetic Regulations Involving the Transcription Factor Specificity Protein 1. Carcinogenesis.

[69] Lam J.B.B., Chow K.H.M., Xu A., Lam K.S.L., Liu J., Wong N.-S., Moon R.T., Shepherd P.R., Cooper G.J.S., Wang Y. (2009). Adiponectin Haploinsufficiency Promotes Mammary Tumor Development in MMTV-PyVT Mice by Modulation of Phosphatase and Tensin Homolog Activities. PLoS ONE.

[70] Sambiasi D., De Summa S., Digennaro M., Pilato B., Paradiso A., Tommasi S. (2017). Adipokines in Hereditary Breast Cancer Patients and Healthy Relatives. Oncotarget.

[71] Naimo G.D., Gelsomino L., Catalano S., Mauro L., Andò S. (2020). Interfering Role of ERα on Adiponectin Action in Breast Cancer. Front. Endocrinol..

[72] Jardé T., Caldefie-Chézet F., Goncalves-Mendes N., Mishellany F., Buechler C., Penault-Llorca F., Vasson M.P. (2009). Involvement of Adiponectin and Leptin in Breast Cancer: Clinical and in Vitro Studies. Endocr. Relat. Cancer.

[73] Chung S.J., Nagaraju G.P., Nagalingam A., Muniraj N., Kuppusamy P., Walker A., Woo J., Győrffy B., Gabrielson E., Saxena N.K. (2017). ADIPOQ/Adiponectin Induces Cytotoxic Autophagy in Breast Cancer Cells through STK11/LKB1-Mediated Activation of the AMPK-ULK1 Axis. Autophagy.

[74] Nakayama S., Miyoshi Y., Ishihara H., Noguchi S. (2008). Growth-Inhibitory Effect of Adiponectin via Adiponectin Receptor 1 on Human Breast Cancer Cells through Inhibition of S-Phase Entry without Inducing Apoptosis. Breast Cancer Res. Treat..

[75] Dieudonne M.-N., Bussiere M., Dos Santos E., Leneveu M.-C., Giudicelli Y., Pecquery R. (2006). Adiponectin Mediates Antiproliferative and Apoptotic Responses in Human MCF7 Breast Cancer Cells. Biochem. Biophys. Res. Commun..

[76] Dos Santos E., Benaitreau D., Dieudonne M.-N., Leneveu M.-C., Serazin V., Giudicelli Y., Pecquery R. (2008). Adiponectin Mediates an Antiproliferative Response in Human MDA-MB 231 Breast Cancer Cells. Oncol. Rep..

[77] Grossmann M.E., Nkhata K.J., Mizuno N.K., Ray A., Cleary M.P. (2008). Effects of Adiponectin on Breast Cancer Cell Growth and Signaling. Br. J. Cancer.

[78] Pham D.-V., Park P.-H. (2022). Adiponectin Triggers Breast Cancer Cell Death via Fatty Acid Metabolic Reprogramming. J. Exp. Clin. Cancer Res..

[79] Fares J., Fares M.Y., Khachfe H.H., Salhab H.A., Fares Y. (2020). Molecular Principles of Metastasis: A Hallmark of Cancer Revisited. Signal Transduct. Target. Ther..

[80] Taliaferro-Smith L., Nagalingam A., Zhong D., Zhou W., Saxena N., Sharma D. (2009). LKB1 Is Required for Adiponectin-Mediated Modulation of AMPK–S6K Axis and Inhibition of Migration and Invasion of Breast Cancer Cells. Oncogene.

[81] Saxena N.K., Sharma D. (2010). Metastasis Suppression by Adiponectin: LKB1 Rises up to the Challenge. Cell Adhes. Migr..

[82] Kim K., Baek A., Hwang J.-E., Choi Y.A., Jeong J., Lee M.-S., Cho D.H., Lim J.-S., Kim K.I., Yang Y. (2009). Adiponectin-Activated AMPK Stimulates Dephosphorylation of AKT through Protein Phosphatase 2A Activation. Cancer Res..

[83] Kang J.-H., Yu B.-Y., Youn D.-S. (2007). Relationship of Serum Adiponectin and Resistin Levels with Breast Cancer Risk. J. Korean Med. Sci..

[84] Adya R., Tan B.K., Chen J., Randeva H.S. (2012). Protective Actions of Globular and Full-Length Adiponectin on Human Endothelial Cells: Novel Insights into Adiponectin-Induced Angiogenesis. J. Vasc. Res..

[85] Chedid P., Hurtado-Nedelec M., Marion-Gaber B., Bournier O., Hayem G., Gougerot-Pocidalo M.-A., Frystyk J., Flyvbjerg A., El Benna J., Marie J.-C. (2012). Adiponectin and Its Globular Fragment Differentially Modulate the Oxidative Burst of Primary Human Phagocytes. Am. J. Pathol..

[86] Ogunwobi O.O., Beales I.L.P. (2006). Adiponectin Stimulates Proliferation and Cytokine Secretion in Colonic Epithelial Cells. Regul. Pept..

[87] Falk Libby E., Liu J., Li Y., Lewis M.J., Demark-Wahnefried W., Hurst D.R. (2016). Globular Adiponectin Enhances Invasion in Human Breast Cancer Cells. Oncol. Lett..

[88] Pham D.-V., Raut P.K., Pandit M., Chang J.-H., Katila N., Choi D.-Y., Jeong J.-H., Park P.-H. (2020). Globular Adiponectin Inhibits Breast Cancer Cell Growth through Modulation of Inflammasome Activation: Critical Role of Sestrin2 and AMPK Signaling. Cancers.

[89] Pang Z.-Y., Wei Y.-T., Shang M.-Y., Li S., Li Y., Jin Q.-X., Liao Z.-X., Cui M.-K., Liu X.-Y., Zhang Q. (2021). Leptin-Elicited PBX3 Confers Letrozole Resistance in Breast Cancer. Endocr. Relat. Cancer.

[90] Delort L., Bougaret L., Cholet J., Vermerie M., Billard H., Decombat C., Bourgne C., Berger M., Dumontet C., Caldefie-Chezet F. (2019). Hormonal Therapy Resistance and Breast Cancer: Involvement of Adipocytes and Leptin. Nutrients.

[91] Macis D., Gandini S., Guerrieri-Gonzaga A., Johansson H., Magni P., Ruscica M., Lazzeroni M., Serrano D., Cazzaniga M., Mora S. (2012). Prognostic Effect of Circulating Adiponectin in a Randomized 2 × 2 Trial of Low-Dose Tamoxifen and Fenretinide in Premenopausal Women at Risk for Breast Cancer. J. Clin. Oncol..

[92] Ozmen H.K., Erdemci B., Askin S., Sezen O. (2017). Carnitine and Adiponectin Levels in Breast Cancer after Radiotherapy. Open Med..

[93] Otvos L. (2019). Potential Adiponectin Receptor Response Modifier Therapeutics. Front. Endocrinol..

[94] Geetha R.G., Krishnankutty Nair Chandrika S., Saraswathy G.G., Nair Sivakumari A., Sakuntala M. (2021). ROS Dependent Antifungal and Anticancer Modulations of Piper Colubrinum Osmotin. Mol. Basel Switz..

[95] Narasimhan M.L., Coca M.A., Jin J., Yamauchi T., Ito Y., Kadowaki T., Kim K.K., Pardo J.M., Damsz B., Hasegawa P.M. (2005). Osmotin Is a Homolog of Mammalian Adiponectin and Controls Apoptosis in Yeast through a Homolog of Mammalian Adiponectin Receptor. Mol. Cell.

[96] Cohen K.E., Katunaric B., Schulz M.E., SenthilKumar G., Young M.S., Mace J.E., Freed J.K. (2022). Role of Adiponectin Receptor 1 in Promoting Nitric Oxide-Mediated Flow-Induced Dilation in the Human Microvasculature. Front. Pharmacol..

[97] Otvos L., Haspinger E., La Russa F., Maspero F., Graziano P., Kovalszky I., Lovas S., Nama K., Hoffmann R., Knappe D. (2011). Design and Development of a Peptide-Based Adiponectin Receptor Agonist for Cancer Treatment. BMC Biotechnol..

[98] Marangoni R.G., Masui Y., Fang F., Korman B., Lord G., Lee J., Lakota K., Wei J., Scherer P.E., Otvos L. (2017). Adiponectin Is an Endogenous Anti-Fibrotic Mediator and Therapeutic Target. Sci. Rep..

[99] Kumar P., Smith T., Rahman K., Thorn N.E., Anania F.A. (2014). Adiponectin Agonist ADP355 Attenuates CCl4-Induced Liver Fibrosis in Mice. PLoS ONE.

[100] Messaggio F., Mendonsa A.M., Castellanos J., Nagathihalli N.S., Gorden L., Merchant N.B., VanSaun M.N. (2017). Adiponectin Receptor Agonists Inhibit Leptin Induced PSTAT3 and in Vivo Pancreatic Tumor Growth. Oncotarget.

[101] Tiliroside, a Glycosidic Flavonoid, Ameliorates Obesity-Induced Metabolic Disorders via Activation of Adiponectin Signaling Followed by Enhancement of Fatty Acid Oxidation in Liver and Skeletal Muscle in Obese-Diabetic Mice-PubMed. https://pubmed.ncbi.nlm.nih.gov/21889885/.

[102] Akimoto M., Maruyama R., Kawabata Y., Tajima Y., Takenaga K. (2018). Antidiabetic Adiponectin Receptor Agonist AdipoRon Suppresses Tumour Growth of Pancreatic Cancer by Inducing RIPK1/ERK-Dependent Necroptosis. Cell Death Dis..

[103] Ramzan A.A., Bitler B.G., Hicks D., Barner K., Qamar L., Behbakht K., Powell T., Jansson T., Wilson H. (2019). Adiponectin Receptor Agonist AdipoRon Induces Apoptotic Cell Death and Suppresses Proliferation in Human Ovarian Cancer Cells. Mol. Cell. Biochem..

[104] Wang S.-J., Wang C., Wang W.-Q., Hao Q.-Q., Liu Y.-F. (2020). Adiponectin Receptor Agonist AdipoRon Inhibits the Proliferation of Myeloma Cells via the AMPK/Autophagy Pathway. Zhongguo Shi Yan Xue Ye Xue Za Zhi.

[105] Sapio L., Nigro E., Ragone A., Salzillo A., Illiano M., Spina A., Polito R., Daniele A., Naviglio S. (2020). AdipoRon Affects Cell Cycle Progression and Inhibits Proliferation in Human Osteosarcoma Cells. J. Oncol..

[106] Otvos L., Knappe D., Hoffmann R., Kovalszky I., Olah J., Hewitson T.D., Stawikowska R., Stawikowski M., Cudic P., Lin F. (2014). Development of Second Generation Peptides Modulating Cellular Adiponectin Receptor Responses. Front. Chem..

[107] Singh A.K., Joharapurkar A.A., Khan M.P., Mishra J.S., Singh N., Yadav M., Hossain Z., Khan K., Kumar S., Dhanesha N.A. (2014). Orally Active Osteoanabolic Agent GTDF Binds to Adiponectin Receptors, with a Preference for AdipoR1, Induces Adiponectin-Associated Signaling, and Improves Metabolic Health in a Rodent Model of Diabetes. Diabetes.

[108] Ma L., Zhang Z., Xue X., Wan Y., Ye B., Lin K. (2017). A Potent Peptide as Adiponectin Receptor 1 Agonist to against Fibrosis. J. Enzyme Inhib. Med. Chem..

[109] A Collagen Domain-Derived Short Adiponectin Peptide Activates APPL1 and AMPK Signaling Pathways and Improves Glucose and Fatty Acid Metabolisms-PubMed. https://pubmed.ncbi.nlm.nih.gov/29991592/.

[110] Anil Kumar S., Hima Kumari P., Shravan Kumar G., Mohanalatha C., Kavi Kishor P.B. (2015). Osmotin: A Plant Sentinel and a Possible Agonist of Mammalian Adiponectin. Front. Plant Sci..

[111] Otvos Jr. L., Kovalszky I., Olah J., Coroniti R., Knappe D., Nollmann F.I., Hoffmann R., Wade J.D., Lovas S., Surmacz E. (2015). Optimization of Adiponectin-Derived Peptides for Inhibition of Cancer Cell Growth and Signaling. Pept. Sci..

[112] Okada-Iwabu M., Yamauchi T., Iwabu M., Honma T., Hamagami K., Matsuda K., Yamaguchi M., Tanabe H., Kimura-Someya T., Shirouzu M. (2013). A Small-Molecule AdipoR Agonist for Type 2 Diabetes and Short Life in Obesity. Nature.

[113] Xiao M., Qu X., Li C., Lv J., Shi Y., Xie K. (2016). AdipoRon for the Treatment of Type 2 Diabetes in Mice and Its Possible Mechanism of the Liver. Zhongguo Ying Yong Sheng Li Xue Za Zhi Zhongguo Yingyong Shenglixue Zazhi Chin. J. Appl. Physiol..

[114] Zhang N., Wei W.-Y., Liao H.-H., Yang Z., Hu C., Wang S.-S., Deng W., Tang Q.-Z. (2018). AdipoRon, an Adiponectin Receptor Agonist, Attenuates Cardiac Remodeling Induced by Pressure Overload. J. Mol. Med. Berl. Ger..

[115] Nicolas S., Debayle D., Béchade C., Maroteaux L., Gay A.-S., Bayer P., Heurteaux C., Guyon A., Chabry J. (2018). Adiporon, an Adiponectin Receptor Agonist Acts as an Antidepressant and Metabolic Regulator in a Mouse Model of Depression. Transl. Psychiatry.

[116] Wang Y., Wan Y., Ye G., Wang P., Xue X., Wu G., Ye B. (2016). Hepatoprotective Effects of AdipoRon against D-Galactosamine-Induced Liver Injury in Mice. Eur. J. Pharm. Sci..

[117] Yamashita T., Lakota K., Taniguchi T., Yoshizaki A., Sato S., Hong W., Zhou X., Sodin-Semrl S., Fang F., Asano Y. (2018). An Orally-Active Adiponectin Receptor Agonist Mitigates Cutaneous Fibrosis, Inflammation and Microvascular Pathology in a Murine Model of Systemic Sclerosis. Sci. Rep..

[118] Sharan K., Mishra J.S., Swarnkar G., Siddiqui J.A., Khan K., Kumari R., Rawat P., Maurya R., Sanyal S., Chattopadhyay N. (2011). A Novel Quercetin Analogue from a Medicinal Plant Promotes Peak Bone Mass Achievement and Bone Healing after Injury and Exerts an Anabolic Effect on Osteoporotic Bone: The Role of Aryl Hydrocarbon Receptor as a Mediator of Osteogenic Action. J. Bone Miner. Res..

[119] Grochowski D.M., Locatelli M., Granica S., Cacciagrano F., Tomczyk M. (2018). A Review on the Dietary Flavonoid Tiliroside. Compr. Rev. Food Sci. Food Saf..

[120] Bećarević M.B., Nikolić B.S., Ignjatović S.D. (2019). Adiponectin: A Therapeutic Target in the Antiphospholipid Syndrome?. Rheumatol. Int..

[121] Ory V., Kietzman W.B., Boeckelman J., Kallakury B.V., Wellstein A., Furth P.A., Riegel A.T. (2018). The PPARγ Agonist Efatutazone Delays Invasive Progression and Induces Differentiation of Ductal Carcinoma in Situ. Breast Cancer Res. Treat..

[122] Maeda N., Takahashi M., Funahashi T., Kihara S., Nishizawa H., Kishida K., Nagaretani H., Matsuda M., Komuro R., Ouchi N. (2001). PPARgamma Ligands Increase Expression and Plasma Concentrations of Adiponectin, an Adipose-Derived Protein. Diabetes.

[123] Aprahamian T., Bonegio R.G., Richez C., Yasuda K., Chiang L.-K., Sato K., Walsh K., Rifkin I.R. (2009). The Peroxisome Proliferator-Activated Receptor Gamma Agonist Rosiglitazone Ameliorates Murine Lupus by Induction of Adiponectin. J. Immunol..

[124] Lebovitz H.E. (2019). Thiazolidinediones: The Forgotten Diabetes Medications. Curr. Diab. Rep..

[125] Andrade M.L., Gilio G.R., Perandini L.A., Peixoto A.S., Moreno M.F., Castro É., Oliveira T.E., Vieira T.S., Ortiz-Silva M., Thomazelli C.A. (2021). PPARγ-Induced Upregulation of Subcutaneous Fat Adiponectin Secretion, Glyceroneogenesis and BCAA Oxidation Requires MTORC1 Activity. Biochim. Biophys. Acta Mol. Cell Biol. Lipids.

[126] Nesti L., Tricò D., Mengozzi A., Natali A. (2021). Rethinking Pioglitazone as a Cardioprotective Agent: A New Perspective on an Overlooked Drug. Cardiovasc. Diabetol..

[127] Catalano S., Mauro L., Bonofiglio D., Pellegrino M., Qi H., Rizza P., Vizza D., Bossi G., Andò S. (2011). In Vivo and in Vitro Evidence That PPARγ Ligands Are Antagonists of Leptin Signaling in Breast Cancer. Am. J. Pathol..

[128] Xu B., Xing A., Li S. (2021). The Forgotten Type 2 Diabetes Mellitus Medicine: Rosiglitazone. Diabetol. Int..

[129] Vilsbøll T., Christensen M., Junker A.E., Knop F.K., Gluud L.L. (2012). Effects of Glucagon-like Peptide-1 Receptor Agonists on Weight Loss: Systematic Review and Meta-Analyses of Randomised Controlled Trials. BMJ.

[130] Knudsen L.B., Lau J. (2019). The Discovery and Development of Liraglutide and Semaglutide. Front. Endocrinol..

[131] Li D., Xu X., Zhang Y., Zhu J., Ye L., Lee K.O., Ma J. (2015). Liraglutide Treatment Causes Upregulation of Adiponectin and Downregulation of Resistin in Chinese Type 2 Diabetes. Diabetes Res. Clin. Pract..

[132] Alanteet A.A., Attia H.A., Shaheen S., Alfayez M., Alshanawani B. (2021). Anti-Proliferative Activity of Glucagon-Like Peptide-1 Receptor Agonist on Obesity-Associated Breast Cancer: The Impact on Modulating Adipokines’ Expression in Adipocytes and Cancer Cells. Dose-Response.

[133] Su J.-R., Lu Z.-H., Su Y., Zhao N., Dong C.-L., Sun L., Zhao S.-F., Li Y. (2016). Relationship of Serum Adiponectin Levels and Metformin Therapy in Patients with Type 2 Diabetes. Horm. Metab. Res..

[134] Duan X., Zhou M., Zhou G., Zhu Q., Li W. (2021). Effect of Metformin on Adiponectin in PCOS: A Meta-Analysis and a Systematic Review. Eur. J. Obstet. Gynecol. Reprod. Biol..

[135] Mallik R., Chowdhury T.A. (2018). Metformin in Cancer. Diabetes Res. Clin. Pract..

[136] Leng W., Pu D., Jiang J., Lei X., Wu Q., Chen B. (2021). Effect of Metformin on Breast Density in Overweight/Obese Premenopausal Women. Diabetes Metab. Syndr. Obes. Targets Ther..

[137] De A., Kuppusamy G. (2020). Metformin in Breast Cancer: Preclinical and Clinical Evidence. Curr. Probl. Cancer.

[138] Najjar S., Allison K.H. (2022). Updates on Breast Biomarkers. Virchows Arch. Int. J. Pathol..

[139] Lubell W.D. (2022). Peptide-Based Drug Development. Biomedicines.

[140] Wang L., Wang N., Zhang W., Cheng X., Yan Z., Shao G., Wang X., Wang R., Fu C. (2022). Therapeutic Peptides: Current Applications and Future Directions. Signal Transduct. Target. Ther..

[141] Lau J.L., Dunn M.K. (2018). Therapeutic Peptides: Historical Perspectives, Current Development Trends, and Future Directions. Bioorg. Med. Chem..

[142] Tan P.H., Tyrrell H.E.J., Gao L., Xu D., Quan J., Gill D., Rai L., Ding Y., Plant G., Chen Y. (2014). Adiponectin Receptor Signaling on Dendritic Cells Blunts Antitumor Immunity. Cancer Res..

[143] Nehme R., Hallal R., El Dor M., Kobeissy F., Gouilleux F., Mazurier F., Zibara K. (2021). Repurposing of Acriflavine to Target Chronic Myeloid Leukemia Treatment. Curr. Med. Chem..

